# Wrist Photoplethysmography Signal Quality Assessment for Reliable Heart Rate Estimate and Morphological Analysis

**DOI:** 10.3390/s22155831

**Published:** 2022-08-04

**Authors:** Serena Moscato, Stella Lo Giudice, Giulia Massaro, Lorenzo Chiari

**Affiliations:** 1 Department of Electrical, Electronic, and Information Engineering “Guglielmo Marconi”—DEI, University of Bologna, 40136 Bologna, Italy; serena.moscato3@unibo.it; 2 School of Engineering (Digital Technology Engineering), Pulsed Academy, Fontys University of Applied Science, 5612 MA Eindhoven, The Netherlands; s.logiudice@fontys.nl; 3 Department of Medical and Surgical Sciences, University of Bologna, 40138 Bologna, Italy; giulia.massaro@unibo.it; 4 Health Sciences and Technologies—Interdepartmental Center for Industrial Research (CIRI-SDV), University of Bologna, 40136 Bologna, Italy

**Keywords:** heart rate, morphological analysis, photoplethysmography, quality assessment, wearable devices

## Abstract

Photoplethysmographic (PPG) signals are mainly employed for heart rate estimation but are also fascinating candidates in the search for cardiovascular biomarkers. However, their high susceptibility to motion artifacts can lower their morphological quality and, hence, affect the reliability of the extracted information. Low reliability is particularly relevant when signals are recorded in a real-world context, during daily life activities. We aim to develop two classifiers to identify PPG pulses suitable for heart rate estimation (Basic-quality classifier) and morphological analysis (High-quality classifier). We collected wrist PPG data from 31 participants over a 24 h period. We defined four activity ranges based on accelerometer data and randomly selected an equal number of PPG pulses from each range to train and test the classifiers. Independent raters labeled the pulses into three quality levels. Nineteen features, including nine novel features, were extracted from PPG pulses and accelerometer signals. We conducted ten-fold cross-validation on the training set (70%) to optimize hyperparameters of five machine learning algorithms and a neural network, and the remaining 30% was used to test the algorithms. Performances were evaluated using the full features and a reduced set, obtained downstream of feature selection methods. Best performances for both Basic- and High-quality classifiers were achieved using a Support Vector Machine (Acc: 0.96 and 0.97, respectively). Both classifiers outperformed comparable state-of-the-art classifiers. Implementing automatic signal quality assessment methods is essential to improve the reliability of PPG parameters and broaden their applicability in a real-world context.

## 1. Introduction

Wearable devices (WDs) are among the most widespread technologies introduced in recent years [1], potentially revolutionizing healthcare. With the aging population and the higher incidence of chronic diseases [2,3], there is a growing need to provide healthcare services capable of reaching people who require frequent medical check-ups, especially those with low mobility and who live in remote areas. With their compact dimensions, high portability, and low manufacturing cost, WDs can efficiently perform long-term recordings outside healthcare facilities, allowing for the remote, continuous monitoring of a user’s health and, in turn, the early detection of anomalies [4,5].

Commonly embedded in commercial smartwatches and fitness trackers worn at the wrist, one of the most used WD technologies is photoplethysmography (PPG), an optical technique that detects blood volume changes using a light source and a matched photodetector. The former illuminates a portion of the body surface, penetrating the skin and blood vessels. The latter detects the changes (using reflected or transmitted light, based on the PPG sensor design [6]) modulated by the pulsatile blood flow, which mainly depends on the heartbeat, vessel stiffness, and respiratory rate [7].

**Figure 1:**
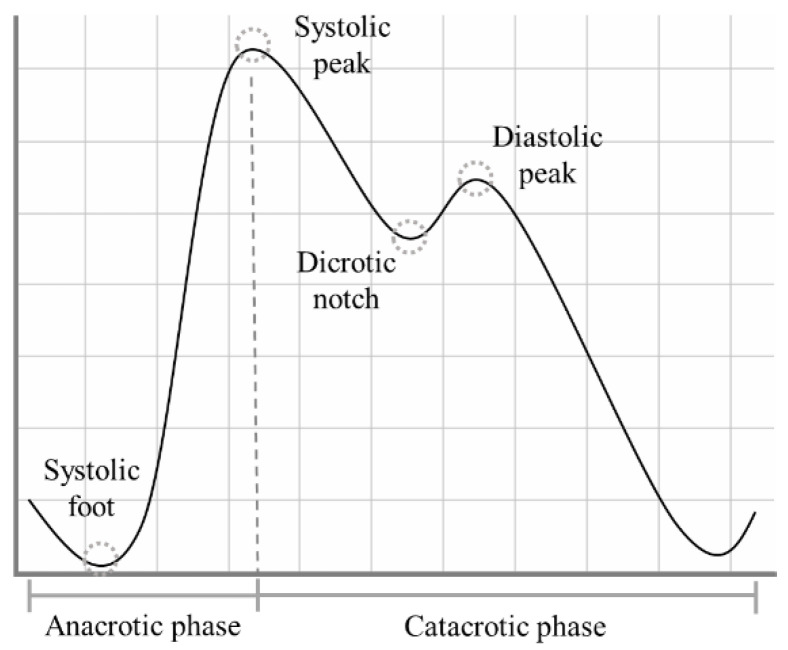
The shape of a typical PPG pulse.

The PPG signal presents a quasi-periodic stereotyped waveform, commonly called PPG pulse, which occurs with each heartbeat [8]. Each PPG pulse can be divided into two phases: the anacrotic phase, which relates to the systolic heart contraction, and the catacrotic phase, which depends both on the diastolic heart phase and on the pulse wave reflected from the peripheral artery [9]. Within each PPG pulse, in ideal conditions, four fiducial points can be identified, as highlighted in Figure 1:

- Systolic foot: the beginning of the systolic phase and the minimum of the pulse;
- Systolic peak: the most prominent maximum;
- Dicrotic notch: most visible in healthy young subjects, it is supposed to represent the closure of the aortic valve [10];
- Diastolic peak: the second prominent maximum of the pulse.

The PPG signal is strictly related to heart dynamics. Indeed, it is extensively used in commercial devices for heart rate (HR) estimation [3,11] and subsequent HR variability (HRV) analysis [12,13]. For example, HR can be estimated simply by detecting the systolic foot or peak, calculating the time difference between two consecutive occurrences, and then calculating the ratio between 60 and the calculated time difference, expressing it in beats/min [14,15].

Besides the HR estimation, it has long been recognized that the PPG signal carries valuable information in its morphology [16]. Recent research has corroborated this finding in emotion recognition [17,18,19] and cardiovascular measurements [20,21].

In real-world applications, the preferred ground for PPG technology, obtaining reliable estimates both for HR and morphological features, is hampered by its high susceptibility to external noise and motion artifacts [22,23]. Consequently, the information above cannot be used in clinical practice for diagnostic purposes. Before further processing, a signal quality analysis is essential to promote this signal’s clinical use.

Based on the definitions provided by the recent literature [2,24], the quality of a PPG pulse exploitable for further analysis can be expressed as:

- Basic-quality pulse: systolic peaks are clearly identifiable;
- High-quality pulse: the pulse waveform is clean and well-defined, with systolic and diastolic waves visible.

While HR and some morphological features related to detecting the systolic peak can be estimated from Basic-quality pulses, more sophisticated morphological features require the detection of both systolic and diastolic peaks [25,26,27], so only High-quality pulses are suitable.

Several researchers have already developed automatic methods for PPG signal quality assessment. Table 1 shows a selection of their works [24,28,29,30,31,32,33,34,35,36,37].

Such studies significantly advanced the development of PPG signal quality algorithms, providing methods that can be used in real time [32,33,34], trained on specific populations [28,31], and validated by making use of publicly available datasets [32,35,36].

However, most previous studies only aim to detect PPG pulses for HR estimate, without rating their suitability for a more in-depth morphological analysis [31,32,33,34,35,36,37]. Moreover, some base the quality estimation on a time window that includes several pulses [24,28,29,31,34,36,37] rather than a pulse-wise analysis, losing relevant information that individual PPG pulses can convey as a result. Such a segment-wise analysis might also discard pulses suitable for analysis.

Although the publicly available datasets represent a considerable resource for training and testing automatic classifiers, they do not allow for a proper quality characterization for real-world purposes. To the best of our knowledge, most of the currently available datasets are based on recordings of finger PPG signals in a clinical context, imposing several limitations. Since it is well-known that the morphology strongly depends on the measurement site [10,38], the translation of a method based on signals recorded at the finger to signals recorded at the wrist (the preferred measurement site for real-world applications) is not feasible. Furthermore, the available datasets do not provide any ground truth information about the different quality of the signals (i.e., Basic and High), but only dichotomous labels (e.g., usable vs. non-usable). Finally, these datasets rely on hospital recordings, a context in which motion artifacts are far less frequent and less impactful than in the real world during daily life activities.

Recent works used PPG signals recorded by wrist-worn WDs in a real-world context and collected PPG pulses prone to lifelike motion artifacts [29,30,31] to overcome these limitations. Unfortunately, in these studies, no information is provided about the motion of the sensors, so it is unclear to what degree the related method is robust to daily life motion artifacts.

This work aimed to develop two motion-aware classifiers:

- Basic-quality classifier: it detects all pulses with valid information content, exploitable for heart rate estimation, and the extraction of basic morphological features;
- High-quality classifier: it detects all pulses with distinct systolic and diastolic waves, exploitable for the extraction of more in-depth morphological features.

We collected wrist PPG data for about 24 h to design and test our classifiers in a real-world context. First, we defined different activity ranges to categorize the level of motor activity, which translates into motion artifacts in the PPG signals. Activity ranges were identified based on data from the accelerometer embedded in the same wrist-worn WD used to record the PPG signal. Then, for each range from each subject, we randomly selected PPG pulses to be classified. In this way, the classifiers could be trained using data subjected to different levels of motion artifacts, usually experienced in real-world contexts.

Such an approach could help in improving the reliability of the valuable biomarkers obtained by wrist PPG signals, minimizing the loss of information by conducting a pulse-wise analysis and selecting pulses suited for a specific analysis (i.e., HRV and fundamental morphological analysis or a more in-depth morphological analysis).

## 2. Materials and Methods

### 2.1. Wearable Device

An Empatica E4 [39] wristband was used to record the signals. The E4 is a CE medical-grade device that allows for the continuous, simultaneous recording of several physiological signals, including PPG and accelerometer data. The PPG sensor is equipped with four light sources (two green, two red) and two photodetectors; the signal is sampled at a frequency of 64 Hz. The tri-axial accelerometer has a range of ±2 g and is sampled at 32 Hz.

### 2.2. Participants

A total of 31 recordings by as many participants were used. All the subjects were instructed to wear the Empatica E4 for 24 h while carrying on with their normal daily activities. The participants were asked to provide their age and gender; other personal information was not collected.

### 2.3. PPG Preprocessing and Pulse Detection

A second-order Butterworth band-pass filter with cut-off frequencies of 0.5 and 12 Hz was applied for each PPG recording [31]. The algorithm by Elgendi et al. [40], originally developed to detect second derivative PPG fiducial points, was adapted to detect the systolic peak and systolic foot of each pulse to segment the signal into single pulses. Each pulse was then normalized with the z-score procedure:

(1) $${\mathrm{pulse}}_{\mathrm{norm}}=\frac{\mathrm{pulse}-\mathrm{mean}\left(\mathrm{pulse}\right)}{\mathrm{std}\left(\mathrm{pulse}\right)}$$

### 2.4. Activity Index and Definition of Activity Ranges

To categorize pulses according to different amounts of movement, the activity index ( $A_{ind}$ ) presented in [41] was calculated for each pulse. To this aim, each accelerometer (ACC) component (*x*, *y, z*) was resampled at $fs_{ACC-RES}\;$ = 64 Hz with linear interpolation (to match the PPG sampling frequency) and converted to *g* units. Next, a fourth-order band-pass filter was applied, with cut-off frequencies of 0.025 and 10 Hz [42,43]. The ACC vector magnitude was then calculated for each sample *j* as:

(2) $$A_{j}=\sqrt{{\mathrm{ACC}}_{x_{j}}{}^{2}+{\mathrm{ACC}}_{y_{j}}{}^{2}+{\mathrm{ACC}}_{z_{j}}{}^{2}}$$

The $A_{ind}$ was estimated using the algorithm of Lin et al. [41]:

- Standard deviation of $A_{j}$ for 5-second epochs:

(3) $$\sigma =\sqrt{\frac{1}{N}\sum_{j=1}^{N}{\left(A_{j}-\mu \right)}^{2}}$$

where

$\mu =\frac{1}{N}\left(A_{1}+A_{2}+\text{…}+A_{N}\right)$

$N=5\;s*fs_{ACC-RES}$

- Minute-wise $A_{ind}$ :

(4) $$A_{ind}=\sum_{k=1}^{M}\sigma_{k}$$

where *M* is set to 12 to obtain a minute-wise $A_{ind}$ by summing 12 5-second epochs.

Once we estimated the $A_{ind}$ for each recording, we defined four activity ranges (AR) based on the quartiles of all the $A_{ind}$ values to label an equal number of pulses in each activity range.

### 2.5. Labelling Procedure

Within each recording, we randomly selected a subset of 100 PPG pulses from each activity range, thus obtaining 400 pulses for each recording (12,400 labelled pulses in total). Three independent raters (S.M., S.L.G., and G.M.) then assigned a quality level to each pulse, selecting from one of the three levels defined below [2]:

- Bad (B): systolic and diastolic peaks cannot be easily distinguished from noise → the pulse is not suitable for further analysis.
- Fair (F): the systolic peak is clearly detectable; the diastolic peak is not → it is possible to estimate the heart rate and some basic morphological features.
- Excellent (E): systolic and diastolic peaks are both clearly detectable → it is possible to estimate the heart rate, and basic morphological features, and perform an in-depth morphological analysis.

**Figure 2:**
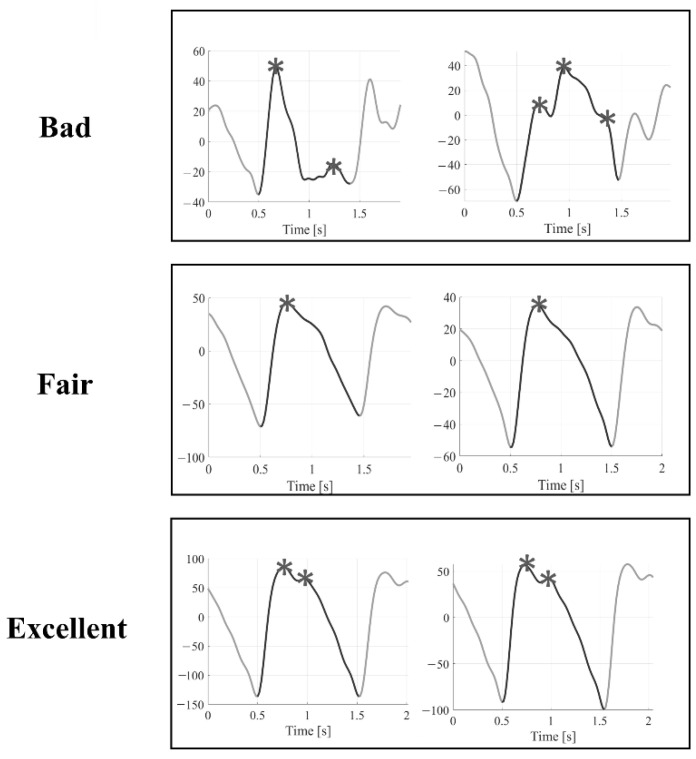
Examples of Bad, Fair, and Excellent quality pulses. Asterisks represent the local maxima for each pulse found by the Matlab *findpeaks* function.

**Figure 3:**
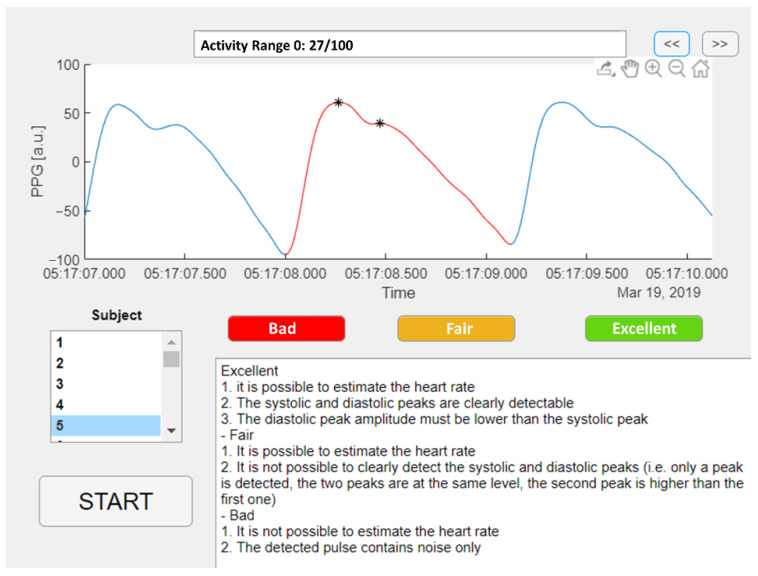
Matlab graphic user interface for PPG pulses annotation.

An example of the three quality levels is illustrated in Figure 2. A Matlab graphic user interface was developed to help the raters annotate the quality of the selected pulses, as shown in Figure 3. The Matlab *findpeaks* function was applied to highlight the local maxima of the selected pulse and help detect the systolic and diastolic peaks.

Inter-rater agreement was assessed by calculating the overall Fleiss Kappa Score [44]. A majority voting approach was applied to determine the level if only two raters agreed. If there was no agreement among raters (i.e., each rater chose a different quality level), the pulse was automatically labelled as B.

### 2.6. Signal Quality Indices

We estimated nineteen signal quality indices (SQIs), listed in Table 2, corresponding to the selected and labelled pulses recorded in a real-world context. Specifically, we estimated:

- 2 SQIs from accelerometer data;
- 17 SQIs from PPG pulses.

We estimated the computational complexity of each feature in terms of Floating-point operations (FLOPs) by using the Matlab package developed by Qian [45].

Labelled PPG pulses were divided into training and test sets, with a proportion of 70% for the training set (22 subjects; 8800 pulses) and 30% for the test set (9 subjects; 3600 pulses).

SQIs from the training and test set pulses were then separately subjected to a Box-Cox transformation [46] and z-scored.

### 2.7. SQIs Selection

To limit the use of redundant SQIs, we applied a Neighborhood Component Analysis (NCA) separately for the two classifiers. NCA is a non-parametric method for selecting features to maximize a classifier’s accuracy [47]. As output, NCA provides a weight for each feature: the higher the weight, the more influential the feature is for solving the classification problem. We first tuned the NCA regularization parameter λ using ten-fold cross-validation on the training set to find the value that minimizes the classification loss. We then labelled those features with a weight greater than 20% of the maximum weight. To reach higher robustness of the selected features set, we ran the NCA ten times and then selected those features that were labelled at least 80% of the time.

### 2.8. Basic- and High-Quality Classifiers

We designed the following classifiers:

- Basic-quality (BQ) classifier: it detects those pulses that can be used to estimate heart rate and for basic morphological analysis (i.e., the union of F and E pulses);
- High-quality (HQ) classifier: it detects those pulses that can be used for in-depth morphological analysis (i.e., E pulses).

To develop the HQ classifier, we investigated two alternative strategies:

1. Discern the union of B and F pulses against E pulses through a single-stage approach;
2. Discern between F and E pulses downstream of a BQ classifier through a multi-stage approach.

**Figure 4:**
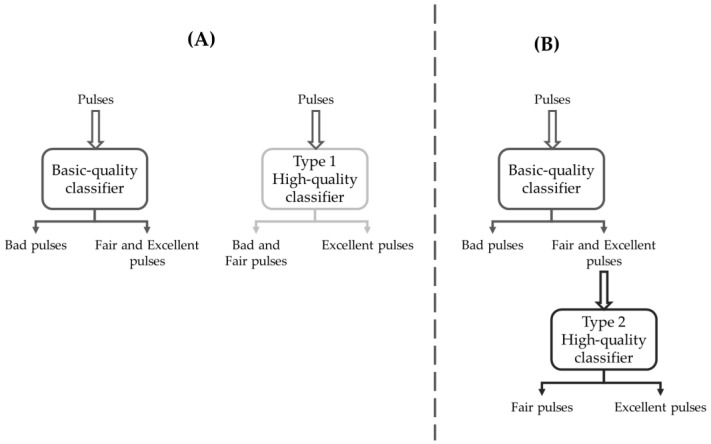
Schematic representation of the classification strategies. (**A**) Two independent classifiers: the Basic-quality classifier aims at detecting Fair and Excellent pulses against Bad pulses, and the Type 1 High-quality classifier aims to detect Excellent pulses against Bad and Fair pulses. (**B**) Cascaded classifiers, with Type 2 High-quality classifier fed with Fair and Excellent pulses selected by the Basic-quality classifier and aimed at detecting Excellent pulses against Fair pulses.

A scheme illustrating the two strategies and the related classifiers is shown in Figure 4. In summary:

- The BQ classifier is trained to detect the F&E classes against the B class;
- The Type 1 HQ classifier (HQ1) is independent of BQ and is trained to detect the E class against the B&F class (Figure 3, panel A);
- The Type 2 HQ classifier (HQ2) is trained to detect the E class against the F class, having as an input the pulses selected by the BQ classifier (Figure 3, panel B).

We first split the dataset into training (70%) and test (30%) sets both for BQ and HQ classifiers. We then conducted a ten-fold cross-validation on the training set with five machine learning (ML) algorithms (Tree, Naïve Bayes, Support Vector Machine, K-nearest neighborhood, and Ensemble) and a neural network (NN) for hyperparameters optimization by using Bayesian optimization with 30 iterations. Finally, we trained and tested the classifiers with the full features set, and the SQIs selected features only.

We computed the following performance metrics on unseen data coming from the test set relative to the detection of eligible pulses (F&E pulses for the BQ classifier, E for HQ classifiers): area under the ROC curve (AUC), accuracy, sensitivity, specificity, precision, Matthew’s correlation coefficient (MCC), F1 score, and Cohen’s kappa (*κ*).

**Figure 5:**
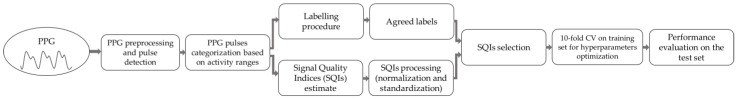
Signal processing and classification pipeline.

All the methods were implemented in Matlab 2021b. The whole signal processing and classification pipeline is illustrated in Figure 5.

### 2.9. State-of-the-Art Classifiers

We selected and adapted two classifiers from the literature to establish a benchmark for the performance of our classifiers.

(i) Jang et al. [30] proposed two classifiers based on the signal similarity between adjacent PPG pulses, a parameter also used in our work (*SigSim*). Their study identified three quality levels (i.e., good, moderate, and low) based on detecting the PPG pulse second derivative’s fiducial points [8]. Then, two dichotomous classifiers, conservative and non-conservative, were developed. The former compares the good-quality level pulses against the merge of moderate- and low-quality level pulses, while the latter compares the good- and moderate-quality level pulses against low-quality level pulses. Each classifier is based on a fixed threshold, determined using the equal training sensitivity and specificity criterion [48], meaning that the optimal threshold is obtained by minimizing the difference between sensitivity and specificity. Jang et al.’s non-conservative classifier is analogous to our BQ classifier, and their conservative classifier is analogous to both our HQ1 and HQ2 classifiers.

(ii) The classifier proposed by Elgendi [24] is built on a Support Vector Machine that classifies 60-second PPG segments as belonging to one of three quality levels (i.e., excellent, acceptable, or unfit for diagnosis) based on the skewness property of the segment. We adapted this method to perform a pulse-wise analysis. Furthermore, since no information regarding the hyperparameters was reported, we applied the same approach described in Section 2.8 to find the best hyperparameters combination.

## 3. Results

### 3.1. Experimental Data

We obtained real-world recordings of physiological signals from 31 subjects (15 males, 16 females), with a mean age of 37 years (±14) and an average recording length of 26:50 h (±05:51). All subjects were Caucasian, except for one African subject.

### 3.2. Activity Ranges

From the $A_{ind}$ values estimated from the accelerometer signal, we obtained the following AR built on the quartile values of the $A_{ind}$ distribution:

- AR_0_: [0–0.0407];
- AR_1_: (0.0407–0.4125];
- AR_2_: (0.4125–1.3254];
- AR_3_: (1.3254 to 6.7474],

According to the classification proposed by Lin et al. [41], the activity ranges 0–3 correspond to rest/sleep, rest/sleep/sedentary, light, and light/moderate activity, respectively. This means that the distribution of $A_{ind}$ is skewed towards lower activity levels in our population.

### 3.3. Labelling Results

**Figure 6:**
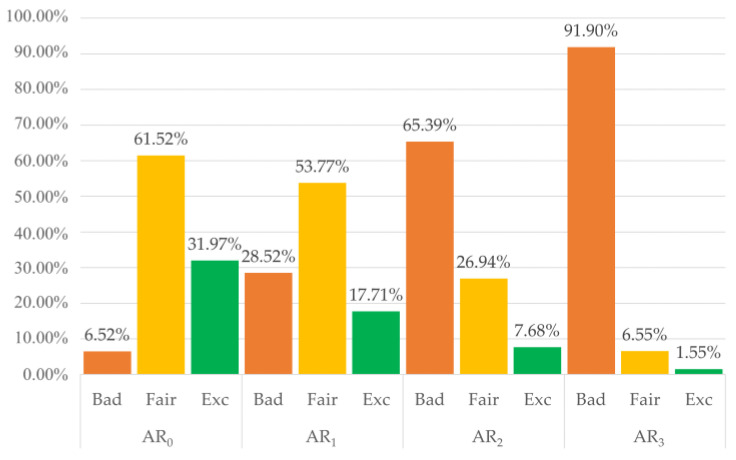
Distribution of the three quality classes among different activity ranges (AR). B = Bad, F = Fair, E = Excellent.

A total of 12,400 pulses were labelled by three independent raters, who agreed on 86% of the labels. Only 57 pulses (0.004%) were labelled differently by each rater and hence relegated to the B category. Overall, the inter-rater agreement was high, with a Fleiss Kappa Score of 0.84, representing perfect agreement according to Landis and Koch [49]. Using a majority voting approach, we set the final labels to train and test the classifiers: 5962 B pulses (48.08%), 4612 F pulses (37.19%), and 1826 E pulses (14.73%). The overall distribution of the three quality levels among the four activity ranges is shown in Figure 6. As expected, as the $A_{ind}$ (the amount of movement) increases, the percentage of B pulses gets higher, and the percentage of F and E pulses gets lower.

### 3.4. SQIs Selection

Considering N, the pulse length, the computational complexity to calculate the 19 features is approximately 37*N FLOPs. The computational complexity for each feature is reported in Supplementary Materials, Table S1.

We conducted SQIs selection separately for the BQ, HQ1, and HQ2 classifiers. In Table 3, the best λ values and their respective minimum classification loss values are reported for the three classifiers.

The selection phase identified eight SQIs for the BQ classifier (*Peak2PeakACC*, *SigSim*, *TroughDepth*, *MedianPulse*, *StdPulse_noZ*, *SNR_Moody, Npeaks*, and *ZDR*), with a computational complexity of approximately 5*N FLOPS, nine SQIs for the HQ1 classifier (*Peak2PeakACC*, *SigSim*, *Kurtosis*, *RelPower*, *Skewness*, *MedianPulse*, *StdPulse_noZ*, *Npeaks*, and *ZDR*), with a computational complexity of approximately 19*N FLOPs, and nine SQIs for the HQ2 classifier (*Entropy, Kurtosis, RelPower, Skewness, MedianPulse, StdPulse_noZ, SNR Moody, Npeaks, and ZDR)*, with a computational complexity of approximately 25*N FLOPs. Results from each iteration of the NCA are reported in the Supplementary Materials, in Tables S2, S3, and S4 for the BQ, HQ1, and HQ2 classifiers, respectively.

### 3.5. Basic-Quality Classifiers

A total of 5962 pulses belong to the B class (4260 used in the training set and 1702 in the test set), while 6438 pulses belong to the F&E class (4540 used in the training set and 1898 in the test set).

Table 4 presents the performances of the BQ classifiers on the test set. The best method using the full features set is the SVM with a Quadratic kernel, reaching an accuracy of 0.9606 and a well-balanced sensitivity (0.9603) and specificity (0.9547). On the other hand, the GentleBoost Ensemble reached the best performance among the methods trained and tested with the selected SQIs, with slightly lower values for accuracy (0.9536) and sensitivity (0.9384) but specificity (0.9706) higher than the best method using the full features set. Final hyperparameters are reported in Supplementary Materials, Table S5.

Concerning the state-of-the-art classifiers, the threshold based on the equal training sensitivity and specificity criterion (identified in the work of Jang et al. [30]) is 0.922. Concerning the classifier proposed by Elgendi [24], the SVM with the Gaussian kernel function provided the best performance in terms of sensitivity (0.8398) and specificity (0.5764) with an accuracy of 0.7153. Our classifier outperformed both state-of-the-art classifiers for the selected performance measures. Results obtained with state-of-the-art classifiers are shown in the lower panel of Table 4.

### 3.6. High-Quality Classifiers

For the Type 1 High-quality classifiers, a total of 10,574 pulses belong to the B&F class (7754 used in the training set and 1702 in the test set), while 1826 pulses belong to the E class (1046 used in the training set and 780 in the test set).

Table 5 presents the performances of the HQ1 classifiers on the test set. The best method for balancing sensitivity and specificity is the SVM, using all the features (Sens = 0.9244, Spec = 0.9784) or the subset of selected SQIs (Sens = 0.9192, Spec = 0.9702). In both cases, the SVM has a Quadratic kernel. Final hyperparameters are reported in Supplementary Materials, Table S6.

For the Type 2 High-quality classifiers, 4612 pulses belong to the F class (3494 used in the training set and 1118 used in the test set), while the distribution of pulses belonging to the E class is the same used to train and test the HQ1 classifiers

Table 6 presents the performances of the HQ2 classifiers on the test set. The kNN method using the subset of features selected by the NCA provided the best results regarding sensitivity–specificity balance (Sens = 0.9321, Spec = 0.9195). The final hyperparameters are reported in Supplementary Materials, Table S7.

By comparing the best HQ1 and HQ2 classifiers, HQ1 achieved better performances in terms of accuracy and specificity (Acc = 0.9667, Spec = 0.9784) with respect to HQ2 (Acc = 0.9247, Spec = 0.9195), but slightly lower sensitivity (HQ1 Sens = 0.9244 vs. HQ2 Sens = 0.9321).

Concerning the state-of-the-art classifiers, the threshold identified for the HQ1 classifier with Jang’s method [30] was 0.991. The linear SVM obtained the best performance in reproducing the classifier proposed by Elgendi [24]. However, both state-of-the-art classifiers performed worse than our classifier: the accuracy was 0.7090 for Jang’s and 0.8406 for Elgendi’s. Notably, the former reached moderate sensitivity (0.6301) and specificity (0.7245), while the latter showed a sensitivity closer to zero (0.0167).

The threshold for the HQ2 classifier with Jang’s method [30] was 0.993. In reproducing Elgendi’s classifier, the quadratic SVM obtained the best performance. Additionally, in this case, both state-of-the-art classifiers performed worse than our best HQ2 classifier, similar to what we observed for the HQ1 classifier.

## 4. Discussion

In this work, we developed automatic classifiers to detect PPG pulses suitable for further processing based on their peculiar morphological characteristics. First, using accelerometer data, we estimated the activity level of the subjects. We then detected four activity ranges based on the quartile values of aggregated $A_{ind}$ s from all the recordings. From each recording, we randomly selected 100 pulses for each activity range. Of the 19 SQIs estimated from each labelled pulse, eight and nine SQIs were selected to train and test the algorithms to develop the Basic- and the two High-quality classifiers, respectively. The best algorithms were then chosen, and the classifiers’ performances were compared against two state-of-the-art classifiers.

Categorizing pulses by activity level allowed us to train the algorithms with pulses containing distinct amounts of motion artifacts. In this way, the ability of classifiers to detect PPG pulses suitable for heart rate estimate or morphological analysis under various movement intensities could be achieved. However, it appears evident from Figure 5 that only a tiny portion of pulses in the highest activity range reached F or E quality levels, even if the highest activity range in our dataset corresponded to light/moderate activity in the staging proposed by Lin et al. [41]. Several methods have been proposed to suppress the effect of motion artifacts on the PPG signals, either via software [50,51] or hardware [52,53] approaches. Our results suggest that future studies should combine algorithms for motion artifact suppression with a layer dedicated to signal quality analysis. This approach would be more conservative, allowing us to obtain reliable parameters from a larger proportion of recorded pulses, even during intense physical activity.

The three independent raters reached a perfect agreement in the labelling procedure, probably thanks to the strict definitions given for each quality level. The high level of the inter-rater agreement also ensures the reliability of the resulting classifiers.

For each PPG pulse, we estimated 19 SQIs, calculated from two sources (i.e., PPG and ACC signals). Nine SQIs were novel and proposed for the first time in this study. The SQIs feature selection phase revealed that eight and nine SQIs were sufficient to solve the classification problem optimally for the BQ and both types of HQ classifiers, respectively. It is worth noting that most of the selected SQIs are novel features. In particular, two of the newly introduced statistical parameters (*MedianPulse*, *StdPulse_noZ*) and two parameters related to the PPG pulse morphology (*Npeaks*, *ZDR*) were selected for all classifiers here presented, adding important information that helped better solve the classification problem.

Although the extraction of multiple features inevitably increases the computational complexity compared with the extraction of a single feature, the cost of the features presented in this work remains low and grows linearly with N. Moreover, it is interesting to note that the NCA selected features with increasing computational complexity for the BQ (5*N FLOPs), HQ1 (19*N FLOPs), and HQ2 (25*N FLOPs) classifiers, in line with the increasing complexity of the classification problem.

It is also worth noting that the *Peak2PeakACC* feature from the accelerometer data was selected only for BQ and HQ1 classifiers, and not for the HQ2 classifier. This can be ascribed to the fact that B pulses (involved in both BQ and HQ1 classifiers) are generated because of motion artifacts, while the F and E pulses are largely independent of the movement.

All the implemented algorithms performed well to achieve BQ and HQ1 classifiers. Except for the Neural Network fed with the full features set, all the methods showed an accuracy higher than 0.90. However, the two classifiers differed in sensitivity and specificity: BQ classifiers showed a balanced sensitivity and specificity, while the HQ classifiers had specificity higher than sensitivity (on average, 0.9728 compared to 0.9729). This difference can be ascribed to the imbalance in the number of pulses in the two classes (only 1826 pulses belonging to the E class compared to 10,574 belonging to the B&F classes), meaning that the algorithms are better trained in detecting pulses belonging to B&F class than to the E class.

Regarding performance, some algorithms used to develop the HQ2 classifiers performed relatively poorly, except for the Ensemble and Tree algorithms. Again, the imbalance between F and E pulses (4612 F pulses against 1826 E pulses) may have played a role. However, as also pointed out by Elgendi [24], it was reasonable to expect that a classifier aiming at detecting E pulses against pulses belonging to a single quality level achieved worse performance than a classifier trained to detect E pulses against different quality pulses. In addition, it is necessary to consider the inevitable error propagation that a system of two cascaded classifiers entails. There may be some B pulses wrongly classified within the F&E pulses by the first stage BQ classifier, so performances might be even worse than the ones reported in this study since the HQ2 classifier was trained and tested only with real F and E pulses.

Our best classifiers outperformed the two state-of-the-art classifiers. Notably, the identified thresholds set for the Jang et al. [30] classifiers were higher than the values reported in the original work: 0.922 versus 0.673 for the BQ classifier, and 0.991 (0.993) versus 0.796 for the HQ1 (HQ2) classifier. These discrepancies could be due to the higher quality levels of the F and E pulses identified in this work. However, the Jang et al. [30] BQ classifier attained good performance, with an accuracy of 0.9253, considering that a single SQI was used. On the other hand, the classifier proposed by Elgendi [24] demonstrated moderate performance for the BQ classifier (Sens = 0.8398, Spec = 0.5764) and poor performance for both HQ classifiers (Sens = 0.0167, Spec = 0.8406 for type 1; Sens = 0, Spec = 0.9991 for type 2).

The proposed classifiers can help extend the use of PPG signals recorded by wearable devices in the real world. On the one hand, the BQ classifier showed promising results, both in terms of sensitivity and specificity. Baek et al. [23] highlighted the detrimental effect on HRV analysis of missing inter-beat intervals. For this reason, a highly sensitive classifier is essential for detecting all pulses that can be used for HR estimation without losing discriminatory power by eliminating too many pulses because of their low quality. On the other hand, SVM selected as the best HQ classifier has high specificity with (relatively) low sensitivity. However, compared to other methods, it shows the best performance in terms of MCC, F1, and Cohen’s κ. The importance of an HQ classifier is obvious, given the number of significant applications that have been proposed in the last few years. Features extracted from PPG morphology could be used, for example, for stress detection purposes [26,54,55] or blood pressure estimation [56,57,58], thus allowing for continuous monitoring with a simple wristband. A large part of the population at risk of developing, e.g., burnout syndromes or cardiovascular disease, would benefit from this achievement.

As a side result of this work, we built an annotated dataset that can be further exploited for future studies. As an ongoing activity, we are working on the preparation of the dataset to be publicly available.

This study has some limitations, most of which are related to the sample population used to train and test the algorithms. First, more robust classifiers could be obtained by increasing the sample size: more subjects and labelled pulses would indeed be beneficial, preferably including subjects with arrhythmias or other cardiac pathologies. As this study was conceived, the classifiers we developed cannot discern arrhythmias from noise, thus potentially discarding arrhythmic beats that could also be useful for diagnostic purposes. Moreover, the algorithms’ training phase could be refined by considering subjects’ age. As pointed out in [7], the dicrotic notch is more pronounced in healthy young than in older adults, and PPG morphology changes with age [25]. Therefore, a future study could collect and balance pulses belonging to different age groups both in the training and testing set. In addition, a further advancement of the method here proposed can be achieved by using recordings from different devices to train the signal quality algorithm. In fact, the results could be device dependent, thus limiting the generalizability to other devices.

The classifiers developed in this study have not been tested in real time. This is a crucial aspect to be assessed to understand whether the signal quality assessment can be smoothly embedded in the processing pipeline of wearable devices to provide reliable information with an acceptable delay [3]. Providing reliable health information in real-time would indeed facilitate the delivery of personalized treatments to the patient if and when needed [59].

## 5. Conclusions

This work aimed to develop two pulse-wise classifiers to detect reliable wrist PPG pulses that can be used in a real-world context for heart rate estimation and morphological analysis. We trained and tested several algorithms with a combination of features derived from different sources, including several novel features, and by selecting PPG pulses subjected to different levels of motion artifacts. The best performances were obtained by using subsets of features for both Basic- and High-quality classifiers. For both classifiers, the SVM with a Quadratic kernel achieved the best performance. Our results could help in improving the reliability and generalizability of the valuable biomarkers obtained by wrist PPG signals. Furthermore, the pulse-wise approach minimizes the loss of information by selecting all pulses suitable for either heart rate variability or morphological analysis. Future work can optimize the classifiers by increasing the sample size (both in terms of subjects and various cardiac health conditions) used to train the algorithms and explore the feasibility of embedding these methods in wearable devices for real-time applications.

## Figures and Tables

**Table 1:** State of the art for the PPG signal quality algorithms.

Ref.	PPG Sensor Position	Settings	# Subjects	Pulse-Wise or Segment-Wise	Ground Truth	Method	# Quality Levels
[28]	Finger and Wrist	Clinical	13 stroke patients + 500 patients retrospectively selected	30 s segments	Labels from 5 raters	Support Vector Machine with 42 features	2 + “not sure”
[29]	Wrist	Real-World	10 elderly subjects + 16 young subjects	10 s segments	Labels from 17 raters	Random forest with 9 features	5
[30]	Wrist	Real-World	50 healthy subjects	Pulse-wise	Labels from 1 rater	Signal similarity between adjacent pulses	3
[31]	Wrist	Real-World	17 epilepsy patients	7 s segments	Correspondence with RR from ECG	Support Vector Machine with PPG and accelerometer features	2
[32]	Finger	Clinical (public DB)	69 subjects from 3 public databases	Pulse-wise	Labels from 2 raters	Rules-based algorithm with 13 quality checks	2
[33]	Finger	Clinical (public DB)	44 patients from 2 public databases	Pulse-wise	Labels from 1 rater	Correlation with a template	2
[24]	Finger	Clinical	40 healthy subjects	60 s segments	Labels from 2 raters	Support Vector Machine with 1 feature	3
[34]	Finger	Clinical (public DB)	No info	10 s segments	Labels from 3 raters	Rules-based algorithm on HR estimate + correlation with a template	2
[35]	Finger	Clinical (public DB)	120 subjects	Pulse-wise	Labels from 1 rater	Non-linear scaling function based on adjacent pulses correlation	2
[36]	Finger	Clinical (public DB)	No info	6 s segments	No info	Deep learning algorithm with 4 features (based on the comparison with a template)	2
[37]	Finger	Lab	13 healthy subjects	60 s segments	Labels from 2 raters	Two-step rules-based algorithm	2

**Table 2:** Signal quality indices (SQIs) for quality classification.

SQI	Description	Source	Ref.
Peak2peakACC	Peak to peak acceleration vector magnitude	ACC	This paper
MeanACC	Mean acceleration vector magnitude	ACC	This paper
SigSim	Correlation between consecutive PPG pulses	PPG	[30]
Entropy	Entropy	PPG	[24]
Kurtosis	Heavy tail and peaked or a light tail and flatness distribution relative to the normal distribution	PPG	[24]
SNR	Signal-to-noise ratio	PPG	[24]
RelPower	Ratio of the power spectral density in the 1–2.25 Hz band compared to the overall power spectral density	PPG	[24]
Skewness	Measure of the symmetry of a probability distribution	PPG	[24]
ZR	Zero-crossing rate	PPG	[24]
Amplitude	Systolic peak amplitude	PPG	[37]
Width	Pulse width	PPG	[37]
TroughDepth	Systolic feet amplitude difference between consecutive systolic feet	PPG	[37]
MedianPulse	Median value of the z-scored PPG pulse	PPG	This paper
MedianPulse_noZ	Median value of the original PPG pulse	PPG	This paper
MeanPulse_noZ	Mean value of the original PPG pulse	PPG	This paper
StdPulse_noZ	Standard deviation of the original PPG pulse	PPG	This paper
SNR_Moody	Signal-to-noise ratio by Moody’s algorithm	PPG	This paper
Npeaks	Number of detected local maxima	PPG	This paper
ZDR	First derivative zero-crossing rate	PPG	This paper

**Table 3:** Final best λ values for neighborhood component analysis and the related minimum classification loss.

	BQ	HQ1	HQ2
Min classification loss	0.0498	0.0395	0.0575
Best λ	0.0017	0.0011	0.016

**Table 4:** Performances for Basic-quality classifiers.

Method	AUC		Acc		Sens		Spec		Prec		MCC		F1		κ	
	All SQIs	Sel. SQIs	All SQIs	Sel. SQIs	All SQIs	Sel. SQIs	All SQIs	Sel. SQIs	All SQIs	Sel. SQIs	All SQIs	Sel. SQIs	All SQIs	Sel. SQIs	All SQIs	Sel. SQIs
Tree	0.9389	0.9413	0.9386	0.9406	0.9331	0.9283	0.9448	0.9542	0.9496	0.9576	0.8771	0.8814	0.9413	0.9428	0.877	0.881
NB	0.9242	0.9227	0.9219	0.92	0.883	0.8725	0.9653	0.973	0.966	0.973	0.8477	0.8455	0.9227	0.92	0.8442	0.8405
SVM	0.9606	0.9519	0.9603	0.9514	0.9547	0.9431	0.9665	0.9606	0.9695	0.9639	0.9205	0.9028	0.962	0.9534	0.9204	0.9026
KNN	0.9497	0.9455	0.9489	0.9453	0.9341	0.942	0.9653	0.9489	0.9678	0.9536	0.8983	0.8904	0.9507	0.9478	0.8977	0.8903
Ensemble	0.9546	0.9545	0.9539	0.9536	0.942	0.9384	0.9671	0.9706	0.9696	.9727	0.9081	0.9078	0.9556	0.9552	0.9077	0.9071
NN	0.9513	0.9511	0.9508	0.9508	0.942	0.9457	0.9606	0.9565	0.9639	0.9604	0.9018	0.9016	0.9528	0.953	0.9015	0.9015
Jang et al. 2018	0.9265		0.9253		0.9025		0.9506		0.9532		0.8519		0.9272		0.8506	
Elgendi 2016	0.7081		0.7153		0.8398		0.5764		0.6886		0.4337		0.7567		0.4215	

**Table 5:** Performances for Type 1 High-quality classifiers (HQ1).

Method	AUC		Acc		Sens		Spec		Prec		MCC		F1		κ	
	All SQIs	Sel. SQIs	All SQIs	Sel. SQIs	All SQIs	Sel. SQIs	All SQIs	Sel. SQIs	All SQIs	Sel. SQIs	All SQIs	Sel. SQIs	All SQIs	Sel. SQIs	All SQIs	Sel. SQIs
Tree	0.9144	0.9217	0.9494	0.9464	0.8526	0.8782	0.9762	0.9652	0.9085	0.8748	0.8484	0.8423	0.8796	0.8765	0.8477	0.8423
NB	0.8838	0.8838	0.9247	0.9283	0.8115	0.8051	0.956	0.9624	0.8362	0.8556	0.776	0.7848	0.8237	0.8296	0.7758	0.7843
SVM	0.9517	0.9447	0.9667	0.9592	0.9244	0.9192	0.9784	0.9702	0.922	0.8951	0.9019	0.881	0.9232	0.907	0.9019	0.8809
KNN	0.8996	0.9234	0.9386	0.9497	0.8308	0.8769	0.9684	0.9699	0.8792	0.8895	0.816	0.8512	0.8543	0.8832	0.8155	0.8511
Ensemble	0.9243	0.9107	0.9614	0.9539	0.859	0.8346	0.9897	0.9869	0.9585	0.9462	0.8839	0.8608	0.906	0.8869	0.8818	0.8581
NN	0.7556	0.9078	0.8881	0.9383	0.5218	0.8538	0.9894	0.9617	0.9314	0.8605	0.6448	0.8178	0.6689	0.8571	0.6078	0.8178
Jang et al. 2018	0.7135		0.7292		0.6859		0.7411		0.4230		0.3685		0.5232		0.3486	
Elgendi 2016	0.5		0.7831		0		0.9906		0		0.0088		NaN		0.00005	

**Table 6:** Performances for Type 2 High-quality classifiers (HQ2).

Method	AUC		Acc		Sens		Spec		Prec		MCC		F1		κ	
	All SQIs	Sel. SQIs	All SQIs	Sel. SQIs	All SQIs	Sel. SQIs	All SQIs	Sel. SQIs	All SQIs	Sel. SQIs	All SQIs	Sel. SQIs	All SQIs	Sel. SQIs	All SQIs	Sel. SQIs
Tree	0.9223	0.8933	0.9278	0.9052	0.891	0.8269	0.9535	0.9597	0.9304	0.9348	0.8505	0.8046	0.9103	0.8776	0.8499	0.8006
NB	0.5	0.5	0.5885	0.5885	0	0	0.9991	0.9991	0	0	0.0192	0.0192	NaN	NaN	0.0011	0.0011
SVM	0.7313	0.9393	0.7713	0.9331	0.5064	0.9744	0.9562	0.9043	0.8896	0.8766	0.5376	0.8679	0.6454	0.9229	0.4948	0.8641
KNN	0.7889	0.9258	0.8145	0.9247	0.6449	0.9321	0.9329	0.9195	0.8702	0.8898	0.6177	0.8462	0.7408	0.9105	0.6013	0.8455
Ensemble	0.9358	0.9191	0.943	0.9273	0.8949	0.8731	0.9767	0.9651	0.9641	0.9458	0.8829	0.8499	0.9282	0.908	0.8812	0.8481
NN	0.5331	0.5258	0.6122	0.6096	0.0885	0.0551	0.9776	0.9964	0.734	0.9149	0.1499	0.1632	0.1579	0.104	0.0762	0.0601
Jang et al. 2018	0.5055		0.5042		0.4397		0.5492		0.405		0.0109		0.4216		0.0108	
Elgendi 2016	0.5		0.5885		0		0.9991		0		0.0192		NaN		0.9204	

## Data Availability

The data presented in this study are available on request from the corresponding author.

## Supplementary Materials

The following supporting information can be downloaded at: https://www.mdpi.com/1424-8220/22/15/5831/s1, Table S1: The computational complexity for each feature. N = pulse length; Table S2: Results from neighborhood component analysis for the Basic-quality classifier applied ten times; Table S3: Results from neighborhood component analysis for the Type 1 High-quality classifier applied ten times; Table S4: Results from neighborhood component analysis for the Type 2 High-quality classifier applied ten times; Table S5: Hyperparameters for Basic-quality classifiers; Table S6: Hyperparameters for Type 1 High-quality classifiers; Table S7: Hyperparameters for Type 2 High-quality classifiers.
