# Supplementary material for: Wrist Photoplethysmography Signal Quality Assessment for Reliable Heart Rate Estimate and Morphological Analysis
Source: Sensors (Basel). 2022 Aug 4;22(15):5831. doi: 10.3390/s22155831 (PMC9370973; doi:10.3390/s22155831)
Supplement: Supplementary file 1 [file sensors-22-05831-s001.zip › sensors-1804747-supplementary.pdf]

Supplementary Materials

Table S1. The computational complexity for each feature. N = pulse length

Feature	Computational complexity
**Peak2peakACC**	1
**MeanACC**	N
**SigSim**	N
**Entropy**	7*N
**Kurtosis**	4*N
**SNR**	8*N
**RelPower**	2*N
**Skewness**	6*N
**ZR**	3
**Amplitude**	1
**Width**	1
**TroughDepth**	1
**MedianPulse**	N
**MedianPulsenoZ**	N
**MeanPulse_noZ**	N
**StdPulse_noZ**	4*N
**SNR_Moody**	8
**Npeaks**	1
**ZDR**	N

Table S2. Results from neighborhood component analysis for the Basic-quality classifier (BQ) applied ten times

Repetition	1	2	3	4	5	6	7	8	9	10	
**Peak2peakACC**	x	x	x	x	x	x	x	x	x	x	**100%**
**MeanACC**											0%
**SigSim**	x	x	x	x	x	x	x	x	x	x	**100%**
**Entropy**											0%
**Kurtosis**						x	x				20%
**SNR**		x				x	x				30%
**RelPower**							x				10%
**Skewness**							x				10%
**ZR**							x				10%
**Amplitude**											0%
**Width**						x	x				20%
**TroughDepth**	x	x	x	x	x	x	x	x	x	x	**100%**
**MedianPulse**		x	x	x	x	x	x		x	x	**80%**
**MedianPulsenoZ**											0%
**MeanPulse_noZ**											0%
**StdPulse_noZ**	x		x	x	x	x	x	x	x	x	**90%**
**SNR_Moody**	x	x	x	x		x	x	x	x	x	**90%**
**Npeaks**	x	x	x	x	x	x	x	x	x	x	**100%**
**ZDR**	x	x	x	x	x	x	x	x	x	x	**100%**

Table S3. Results from neighborhood component analysis for the Type 1 High-quality classifier (HQ1) applied ten times

**Repetition**	**1**	**2**	**3**	**4**	**5**	**6**	**7**	**8**	**9**	**10**	
**Peak2peakACC**	x		x	x	x	x	x	x	x	x	**90%**
**MeanACC**											0%
**SigSim**	x	x	x	x	x	x	x	x	x	x	**100%**
**Entropy**											0%
**Kurtosis**	x		x	x	x	x	x	x	x	x	**90%**
**SNR**	x		x	x	x		x		x		60%
**RelPower**	x	x	x	x	x	x	x	x	x	x	**100%**
**Skewness**	x	x	x	x	x	x	x	x	x	x	**100%**
**ZR**											0%
**Amplitude**											0%
**Width**											0%
**TroughDepth**	x		x	x	x						40%
**MedianPulse**	x		x	x	x	x	x	x	x	x	**90%**
**MedianPulsenoZ**											0%
**MeanPulse_noZ**											0%
**StdPulse_noZ**	x	x	x	x	x	x	x	x	x	x	**100%**
**SNR_Moody**			x	x	x	x	x		x	x	70%
**Npeaks**	x	x	x	x	x	x	x	x	x	x	**100%**
**ZDR**	x	x	x	x	x	x	x	x	x	x	**100%**

Table S4. Results from neighborhood component analysis for the Type 2 High-quality classifier (HQ2) applied ten times

**Repetition**	**1**	**2**	**3**	**4**	**5**	**6**	**7**	**8**	**9**	**10**	
**Peak2peakACC**		x					x				20%
**MeanACC**		x	x			x	x		x		50%
**SigSim**		x				x	x		x		40%
**Entropy**	x	x	x	x	x	x	x	x	x	x	**100%**
**Kurtosis**	x	x	x	x	x	x	x	x	x		**90%**
**SNR**		x					x		x		30%
**RelPower**	x	x	x	x	x	x	x	x	x	x	**100%**
**Skewness**	x	x	x	x	x	x	x	x	x	x	**100%**
**ZR**		x					x				20%
**Amplitude**		x					x		x		30%
**Width**		x					x		x		30%
**TroughDepth**											0%
**MedianPulse**	x	x	x	x	x	x	x	x	x		**90%**
**MedianPulsenoZ**		x					x		x		30%
**MeanPulse_noZ**		x									10%
**StdPulse_noZ**	x	x	x	x	x	x	x	x	x	x	**100%**
**SNR_Moody**	x	x	x	x	x	x	x	x	x	x	**100%**
**Npeaks**	x	x	x	x	x	x	x	x	x	x	**100%**
**ZDR**	x	x	x	x	x	x	x	x	x	x	**100%**

Table S5. Hyperparameters for the Basic-quality classifiers

Algorithms	Hyperparameters	All features	SQIs selection
Tree	Maximum number of splits	39	38
	Split criterion	Gini's diversity index	Gini's diversity index
Naïve Bayes (NB)	Distribution names	Kernel	Kernel
	Kernel type	Gaussian	Gaussian
Support Vector Machine (SVM)	Kernel function	Quadratic	Gaussian
	Kernel scale	1	27.494
	Box constraints	0.025078	119.112
	Standardize data	True	False
K-nearest neighborhood (KNN)	Number of neighbors	10	5
	Distance metrics	Correlation	Chebyshev
	Distance weight	Inverse	Squared inverse
	Standardize data	True	False
Ensemble	Ensemble method	GentleBoost	Bag
	Maximum number of splits	21	1025
	Number of learners	400	285
	Learning rate	0.0093026	-
Neural Network	Number of fully connected layers	2	1
	Activation function	Sigmoid	Tanh
	Regularization strength	1,18E-06	5,45E-05
	Standardize data	No	No
	1st layer size	5	3
	2nd layer size	204	-
	3rd layer size	-	-
Elgendi 2016 (SVM)	Kernel function	Gaussian	
	Kernel scale	0.48469	
	Box constraints	0.015755	
	Standardize data	True	

Table S6. Hyperparameters for the Type 1 High-quality classifiers

Algorithms	Hyperparameters	All features	SQIs selection
Tree	Maximum number of splits	39	38
	Split criterion	Gini's diversity index	Gini's diversity index
Naïve Bayes (NB)	Distribution names	Kernel	Kernel
	Kernel type	Gaussian	Gaussian
Support Vector Machine (SVM)	Kernel function	Quadratic	Gaussian
	Kernel scale	1	27.494
	Box constraints	0.025078	119.112
	Standardize data	True	False
K-nearest neighborhood (KNN)	Number of neighbors	10	5
	Distance metrics	Correlation	Chebyshev
	Distance weight	Inverse	Squared inverse
	Standardize data	True	False
Ensemble	Ensemble method	GentleBoost	Bag
	Maximum number of splits	21	1025
	Number of learners	400	285
	Learning rate	0.0093026	-
Neural Network	Number of fully connected layers	2	1
	Activation function	Sigmoid	Tanh
	Regularization strength	1,18E-06	5,45E-05
	Standardize data	No	No
	1st layer size	5	3
	2nd layer size	204	-
	3rd layer size	-	-
Elgendi 2016 (SVM)	Kernel function	Gaussian	
	Kernel scale	0.48469	
	Box constraints	0.015755	
	Standardize data	True	

Table S7. Hyperparameters for the Type 2 High-quality classifiers

Algorithms	Hyperparameters	All features	SQIs selection
Tree	Maximum number of splits	69	53
	Split criterion	Gini's diversity index	Gini's diversity index
Naïve Bayes (NB)	Distribution names	Gaussian	Gaussian
	Kernel type	Epanechnikov	Triangle
Support Vector Machine (SVM)	Kernel function	Linear	Quadratic
	Kernel scale	1	1
	Box constraints	0.8367	55.859
	Standardize data	False	False
K-nearest neighborhood (KNN)	Number of neighbors	3	39
	Distance metrics	City block	Chebyshev
	Distance weight	Squared inverse	Squared inverse
	Standardize data	False	False
Ensemble	Ensemble method	GentleBoost	Bag
	Maximum number of splits	494	223
	Number of learners	116	480
	Learning rate	0.44184	-
Neural Network	Number of fully connected layers	3	1
	Activation function	Tanh	Sigmoid
	Regularization strength	4,02E-06	2,81E-09
	Standardize data	False	False
	1st layer size	2	4
	2nd layer size	1	-
	3rd layer size	175	-
Elgendi 2016 (SVM)	Kernel function	Quadratic	
	Kernel scale	1	
	Box constraints	0,0017795	
	Standardize data	False	
